# Create Healthy Bodies

**Published:** 1919-01

**Authors:** 


### Create Healthy Bodies.

In a recent address to the Houston High School, Dr. Strayer, of Teachers College, Columbia University, said that the public schools need a "real health service," in the place of the present system of reporting the number of defectives. What he would have is reports as to the number of defectives corrected rather than the number found. He would have the schools profit by the experience gained in the training schools during the war, and have them graduate students healthy in body as well as strong in mind, a good body and good mind making the ideal citizen.

